# Characterization of spiny lobsters from Bangladesh waters using morphology, COI and 16S rRNA sequences

**DOI:** 10.1016/j.heliyon.2022.e08846

**Published:** 2022-01-28

**Authors:** Md. Sagir Ahmed, Anindita Barua, Sujan Kumar Datta, Tonmoy Saha, Durjoy Raha Antu, Sumaiya Ahmed

**Affiliations:** a Department of Zoology, University of Dhaka, Dhaka 1000, Bangladesh; b Department of Genetic Engineering and Biotechnology, University of Dhaka, Dhaka 1000, Bangladesh; c Department of Zoology, Jagannath University, Dhaka 1100, Bangladesh

**Keywords:** Spiny lobster, COI, 16S rRNA, DNA Barcoding, Bangladesh

## Abstract

This study aims to taxonomically identify and characterise the phylogenetic relationships of spiny lobsters based on mitochondrial cytochrome c oxidase I (COI) and 16S rRNA genes from Bangladesh waters. A total of 19 barcode sequences (10 partial COI sequences and 9 partial 16S rRNA) were successfully generated from 12 collected spiny lobster samples representing four species belonging to the family Palinuridae. The average genetic distances within and between species were 0.834 ± 0.427 and 17.810 ± 0.830, respectively, in COI and 0.107 ± 0.255 and 8.401 ± 2.547, respectively, in 16S rRNA genes. The successful amplification rate of 16S rRNA was higher than that of the COI marker. In the maximum likelihood (ML) tree, the sequences of the same species were clustered together under a single clade for both COI and 16S rRNA, which supports the efficacy of both marker genes in differentiating lobster species.

## Highlights

• Four Lobster species, *Panulirus homarus, P. ornatus, P. polyphagus* ​and ​*P. versicolor,* ​were morphologically described.
• Molecular characterization was confirmed based on two markers, COI and 16S rRNA.
• This is the first comprehensive taxonomic description of spiny lobsters from Bangladesh.

## 1 Introduction

Lobsters are one of the most valuable and highly priced crustaceans in domestic and international markets. There are approximately 149 species of lobsters around the world (Holthuis, 1991). Most lobsters fall under one of the two families, Palinuridae, known as spiny lobsters, and Scyllaridae, known as slipper lobsters. The family Palinuridae consists of 47 species of the genus *Palinurus* (Holthuis, 1991), and the Scyllaridae family includes 88 species, 19 genera and two subspecies worldwide (Holthuis, 1991; Carpenter and Niem, 1998; Chan, 2010; Yang et al., 2011; Yang and Chan, 2012). Only four species of spiny lobsters (*Panulirus homarus, P. ornatus, P. polyphagus* and *P. versicolor*) and two species of slipper lobsters (*Thenus orientalis* and *Scyllarus depressus)* have been documented thus far from Bangladesh under the families Palinuridae and Scyllaridae, respectively (Ahmed et al., 2008; IUCN Bangladesh, 2015). Although *Scyllarus depressus* is a Western Atlantic species, it has been recorded from shallow rocky substrates and coral reefs of St. Martins Island, Naf River mouth (Teknaf) and the coast of Bangladesh (Ahmed et al., 2008; IUCN Bangladesh, 2015). Slipper lobster species, on the other hand, are bottom-dwelling lobster species that prefer sandy and muddy habitats and rest in extremely shallow water, as reported off the coast of Cox's Bazar (Carpenter and Niem, 1998; IUCN, 2015). Due to the high market price, spiny lobsters are more highly exploited than slipper lobsters in Bangladesh.

Holthuis (1991) explored the morphological characteristics of adult lobsters in detail of nearly all marine lobsters known up to the early 1990s. Chan (2010) updated the valid species list with several newly described taxa and organized all living marine lobsters into four infraorders: Astacidea, Glypheidea, Achelata and Polychelida. He has recognized six families, 55 genera, 248 species and four subspecies of marine lobsters. However, DNA barcoding develops traditional taxonomy methods and is useful for distinguishing cryptic or polymorphic species of marine lobsters (Chow et al., 2006); moreover, it also allows us to discriminate the species from processed and commercial products (Jeena et al., 2016).

DNA barcoding is a universal method designed to identify species by using partial sequences of the mitochondrial cytochrome c oxidase subunit I (COI) gene (Hebert et al., 2003). The high mutation rate of the gene between interspecific sequences and conserved regions among conspecifics can distinguish even closely related species (Hebert et al., 2003). Another mitochondrial marker, 16S ribosomal RNA, is also considered a candidate marker. As a conserved gene, it can reliably measure the true divergences between closely related organisms. It can be easily amplified and successfully sequenced across various animals, distinguishing specific species (Sinniger et al., 2008; Zheng et al., 2013; Chakraborty and Iwatsuki, 2006; Lee et al., 2014; Vences et al., 2005). The combination of conserved and variable regions within the same gene makes 16S rRNA one of the most popular genes for reconstructing animal phylogenies.

There is no published work thus far on the detailed taxonomic description and molecular characterization of spiny lobsters from Bangladesh. The present study aims to validate the morphologically identified lobster species based on mitochondrial cytochrome c oxidase I (COI) and the 16S rRNA gene.

## 2 Materials and methods

### 2.1 Sampling and morphological analysis

The target lobster specimens were collected as dead from Teknaf (20°46′37.6″N 92°15′20.0″E), Cox's Bazar (21°24′15.3″N 91°53′10.1″E), and St. Martin Island (20°36′49.3″N 92°19′51.6″E) of Bangladesh from December 2020 to March 2021. Immediately after collection, the specimens were preserved in ice and transported to the laboratory for morphological identification. Taxonomic identification of the specimen was performed based on morphometric and meristic characteristics following the guidelines of Ahmed et al. (2008), Carpenter and Niem (1998), and Burton and Davie (2007). Tissue samples were excised and stored in 90% ethanol. Voucher specimens were fixed with 10% formalin and then transferred to 70% ethanol solution for preservation. The species were tagged with a Dhaka University Zoology Museum (DUZM) voucher ID and kept in the institutional museum.

### 2.2 Genomic DNA extraction and amplification by PCR

DNA was extracted from a 5-mg tissue sample collected from the lower abdomen of the specimen using a Promega Wizard® Genomic DNA Purification kit. The quality and quantity of the extracted DNA were measured using a NanoDrop™ spectrophotometer. COI and 16S rRNA gene sequences were amplified by polymerase chain reaction with the primers LCO-1490 (forward) 5′-TCAACAAATCATAAGGACATTGG-3′ and HCO-2198 (reverse) 5′- TAAACTTCAGGGTGTCCAAAGAATCA-3′ for COI (Folmer et al., 1994) and the primers 16Sar (forward) 5′-CGCCTGTTTATCAAAAACAT-3′ and 16Sbr (reverse) 5′- CCGGTCTGAACTCAGATCATGT-3′ for 16S rRNA (Palumbi et al., 1991) genes, respectively. PCR was conducted in 25-μl volumes containing 23 μl of PCR Master Mix (12.5 μl GoTaq® Green Master Mix, 8.5 μl of Nano Pure water, 1 μl of forward primer and 1 μl of reverse primer) and 100 ng of DNA sample and mixed and spun for 30 s for homogenization of the mixture. The amplification conditions included initial denaturation at 95 °C for 5 min followed by 35 cycles of 94 °C for 45 s, 42 °C (COI) and 50 °C (16S rRNA) for 30 s, 72 °C for 45 s, and a final extension at 72 °C for 10 min. The PCR products were kept at room temperature for 15 min and then stored at -26 °C until further downstream application. Amplified gene bands were visualized in a 1% agarose gel and purified using a PureLink™ PCR purification kit. The purified PCR products with DNA concentrations >10 ng/μl were sent to First BASE laboratories, Malaysia, for sequencing. Sequencing was performed by Sanger dideoxy sequencing technology using an ABI PRISM 3730xl Genetic Analyser exploiting the BigDye R Terminator v3.1 cycle sequencing kit chemistry.

### 2.3 Bioinformatics analysis

The assembled contigs of the gene sequences were prepared by the CAP3 DNA assembly program using Unipro Ugene (Okonechnikov et al., 2012). Each sequence was confirmed via BLASTn against the best match sequences of the nucleotide database (identity cut off ≥99%) and deposited in NCBI GenBank. Our analysis includes sequences of the collected species, along with sequences of the identical species retrieved from the NCBI GenBank database. All the COI and 16S rRNA sequences were aligned automatically using MUSCLE (Edgar, 2004). For the distance-based method, genetic pairwise divergence was determined by calculating the Kimura-2-parameter (K2P) (Kimura, 1980) distance using MEGA X (Kumar et al., 2018). The genetic divergence within and between species was illustrated as a box plot distribution using RStudio (RStudio Team, 2015). Phylogenetic trees were constructed for COI and 16S rRNA sequences using Mega X based on the maximum likelihood (ML) statistical method and K2P substitution model with gamma distribution rates. The robustness of clustering was determined by bootstrap analysis with 1000 replicates.

## 3 Results

A total of 12 specimens were examined during the study from the Bangladesh coast: Teknaf, Cox's Bazar and St. Martin's Island. Morphometric identification and molecular characterization confirmed four species of spiny lobsters, *Panulirus homarus, P. ornatus, P. polyphagus* and *P. versicolor.*

### 3.1 Morphological analyses

#### 3.1.1 *Panulirus homarus* (Linnaeus, 1758)

**English Name:** Scalloped spiny lobster

**Material examined:** Two females, Bangladesh, Teknaf, the southernmost point in mainland Bangladesh. 20°46′37.6″N 92°15′20.0″E, ID DUZM_CR_144B- DUZM_CR_144B.2

**Habitat:** Inhabits shallow waters among rocks, often in the surf zone. Maximum depth of 90 m (Chan, 1998).

**Figure 1:**
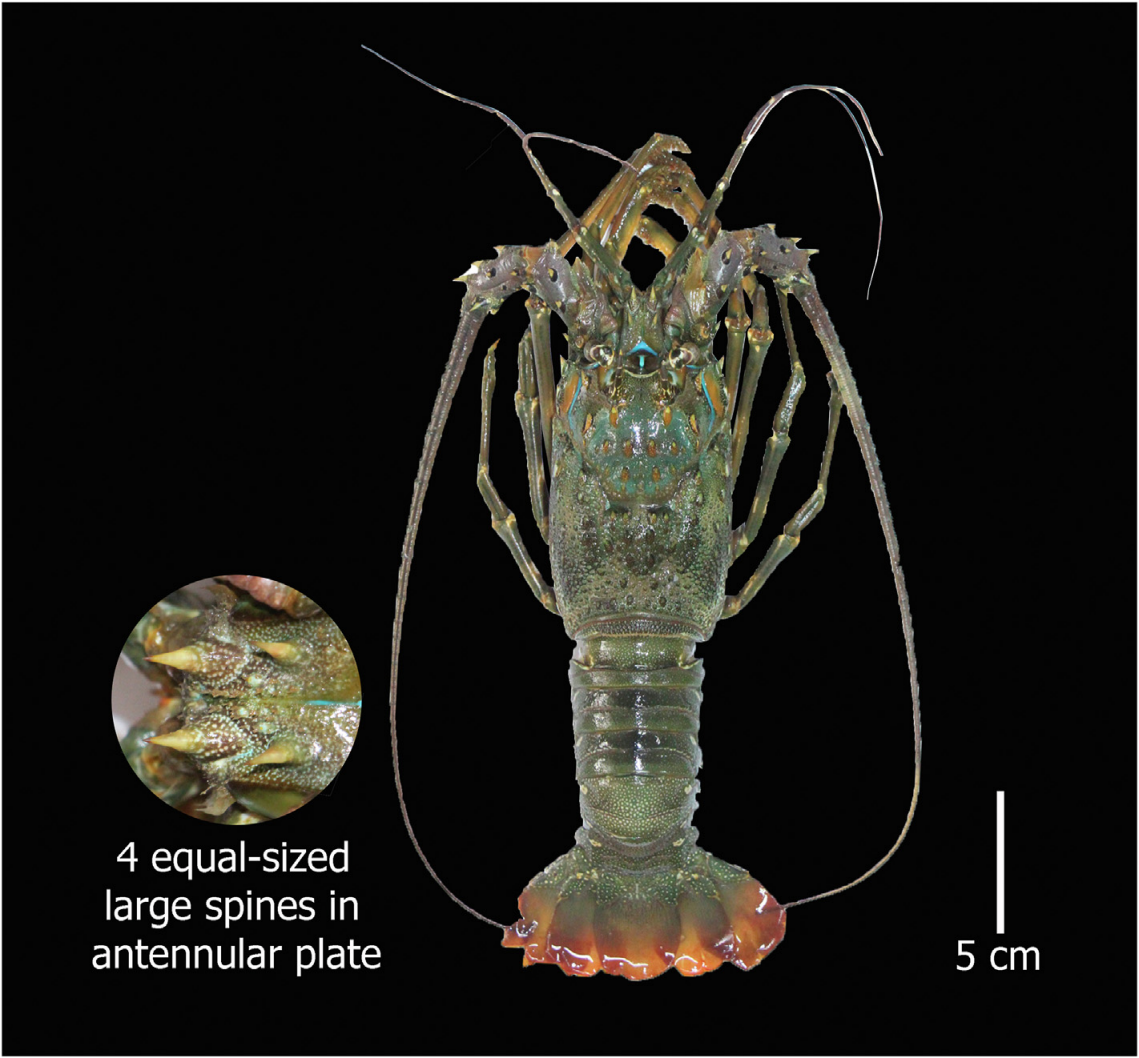
*Panulirus homarus*, Family Palinuridae, voucher ID DUZM_CR_144B, collection date: 28-November-2020, place: Teknaf.

**Characteristics:** Round shape carapace having numerous spines of different sizes; antennular peduncles are smaller than antennular flagella; absence of rostrum; anterior margin armed with 4 frequently spaced large spines apart from the frontal horns; height of the eye is 2 times smaller than the height of the frontal horn; antennular plate bearing 4 properly separated principal spines and few small spinules; every abdominal segment with a transverse groove, occasionally interrupted in the centre, its anterior margins formed into superficial scallops; legs 1 to 4 are without pincers (Figure 1).

**Colour:** Carapace is darkish green to reddish brown in colour. Very small white spots were present on the head and especially distinct on the posterior half of the abdomen. All the legs are darkish green in colour. The stripes of the leg are coloured white. A white and green colour band is present on the antennules.

#### 3.1.2 Panulirus ornatus (Fabricius, 1798)

**English Name:** Ornate spiny lobster

**Material examined:** One female, Bangladesh, St. Martin's Island, northeastern part of the Bay of Bengal. 20°36′49.3″N 92°19′51.6″E, ID DUZM_CR_142

**Habitat:** Inhabits shallow, sometimes slightly turbid coastal waters usually on sand and mud substrates but also on coral reefs and rocky bottoms. Maximum depth of 8 m (George, 1968; Chan, 1998).

**Figure 2:**
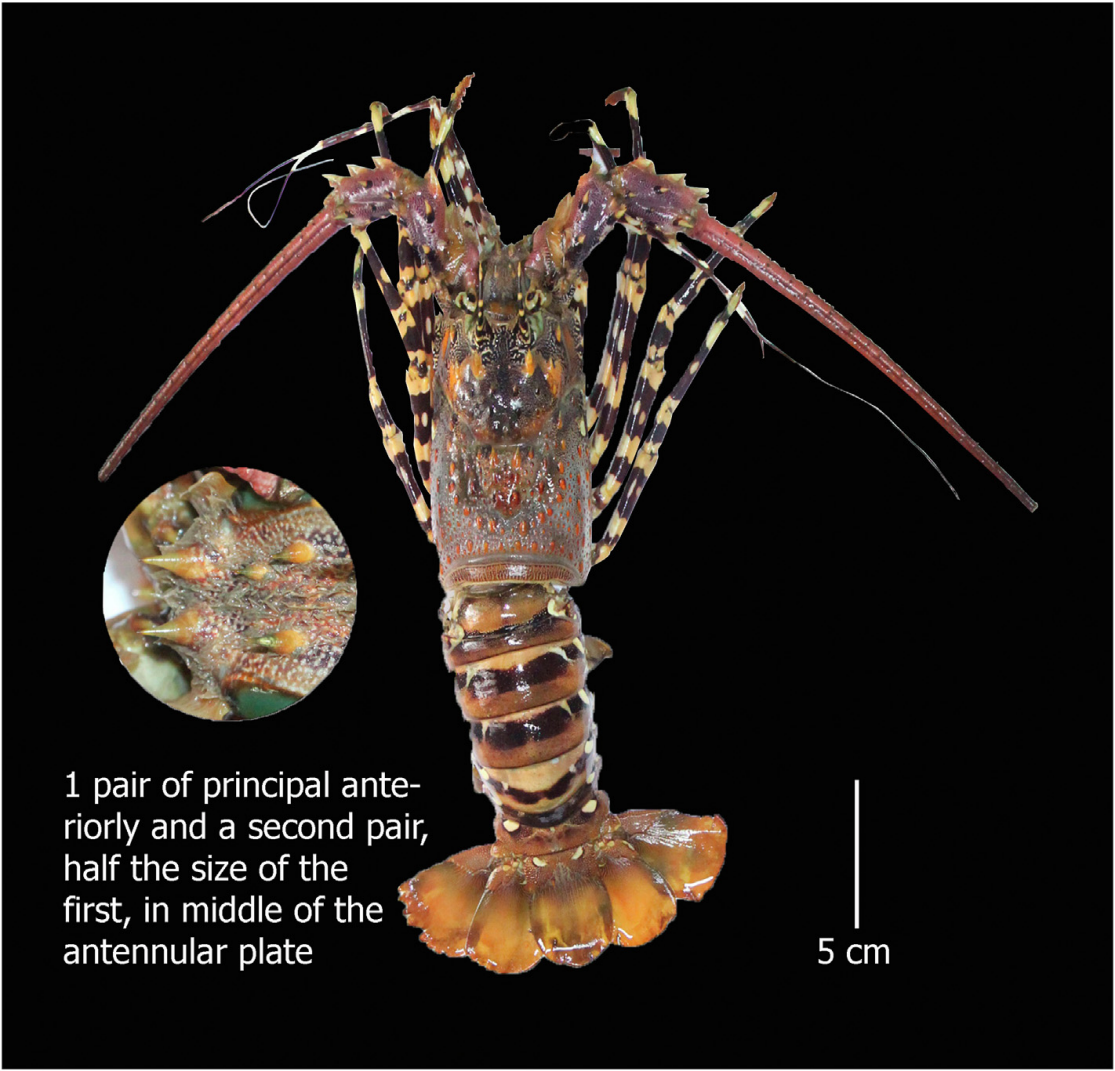
*Panulirus ornatus*, family Palinuridae, voucher ID DUZM_CR_142, collection date: 2-March-2021, place: St. Martin's Island.

**Characteristics:** Carapace rounded and covered with several spines and tubercles of various sizes; absence of rostrum, and antennular peduncles are smaller than antennular flagella; a broad antennular plate separates the bases of the antennae. The antennar plate has a pair of principal spines anteriorly and a second pair that are half the size of the first and a small spine in between two pairs at the right side of the plate (Figure 2). Smooth abdominal segment without a transverse groove; pincers are absent in the legs.

**Colour:** On the yellow carapace, bluish or greenish spines are present. The anterior part of the carapace possesses a vermicular pattern of pale and dark lines near the bases of the frontal horns and the anterior spines. The abdomen has a broad, dark transverse band over the middle of the segments. On the sides, each segment with a large pale spot and an extra elongate mark higher up on the 2^nd^, 3^rd^ and 4^th^ segments at an angle. Transverse white bands are not present along the posterior margin of the segments. Brightly coloured flagella are present and anteriorly banded. Legs having distinct, sharply defined dark and pale blotches.

#### 3.1.3 Panulirus polyphagus (Herbst, 1793)

**English Name:** Mud spiny lobster

**Material examined:** Three females and two males; Bangladesh, Cox's Bazar, southeastern coast of Bangladesh; 21°24′15.3″N 91°53′10.1″E and Bangladesh, St. Martin's Island, northeastern part of the Bay of Bengal. 20°36′49.3″N 92°19′51.6″E; ID DUZM_CR_143- DUZM_CR_143.5

**Habitat:** Mainly found on muddy bottoms (sometimes also on rocky bottoms) in turbid waters near river mouths at depths from 3 to 90 m but usually less than 40 m deep (Morgan, 1980; Holthuis, 1991).

**Figure 3:**
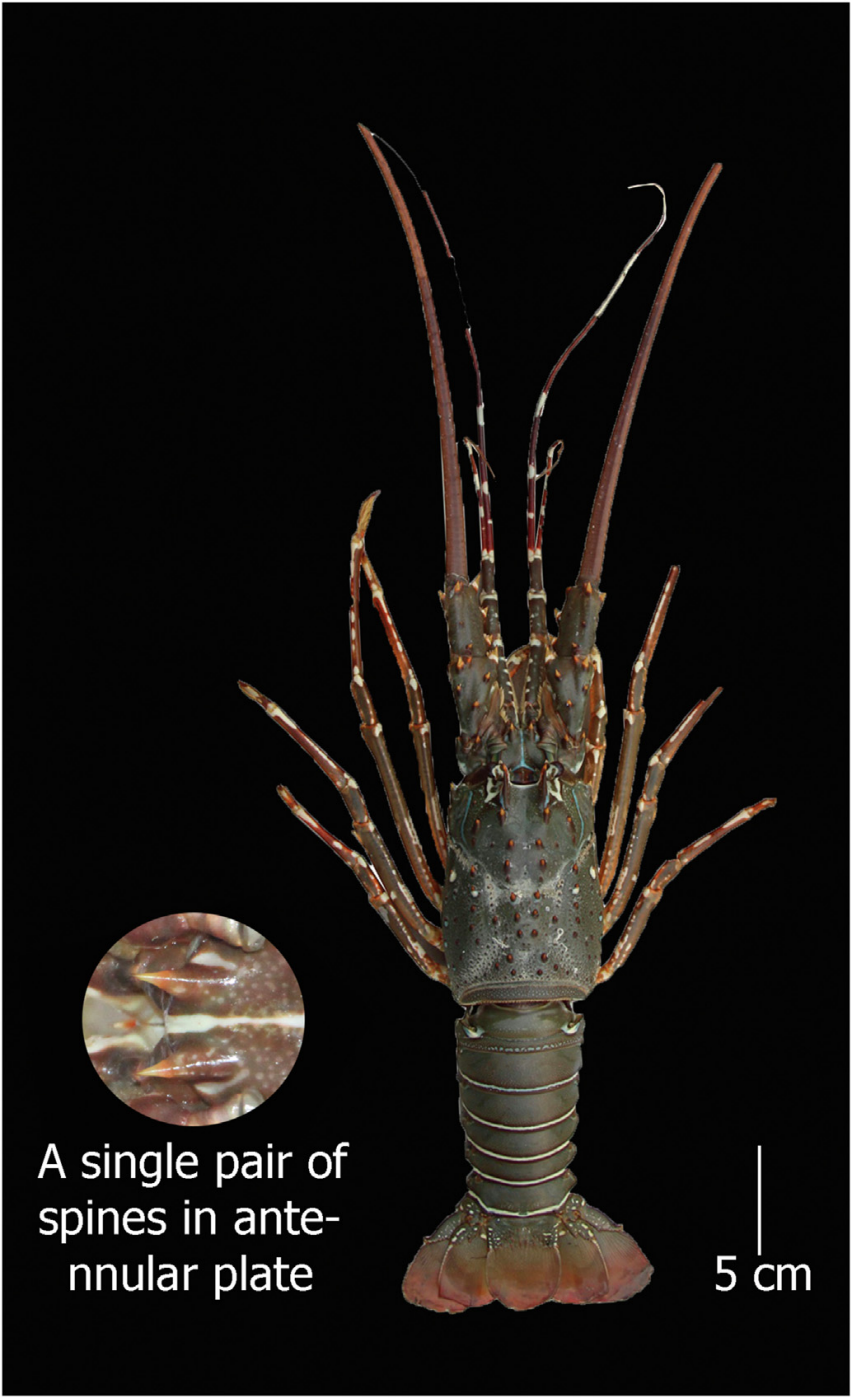
*Panulirus polyphagus*, Family Palinuridae, voucher ID DUZM_CR_143.3, collection date: 17-January-2020, place: Cox's Bazar.

**Characteristics:** Round shape carapace having numerous spines of different sizes; the height of the eye is 2 times larger than the height of the frontal horn; antennular peduncles are smaller than antennular flagella; absence of rostrum; a broad antennular space bearing a single pair of principal spines separates the bases of antennae; no transverse grooves on the abdominal segments; 1 to 4 pereiopods without pincers (Figure 3).

**Colour:** Body dull green in colour, carapace with yellowish brown spines, and eyes are black–brown. The orbital margin and posterior marginal groove are yellowish white in colour. Antennular peduncle alternated with yellowish white and pale green bands. Yellowish white and dark brown bands are present on the flagella. Pereiopods have yellowish white blotches and are a light brown colour. The abdomen is covered by small pale dots. Each abdominal segment has a yellowish white band with brown margins near the posterior border.

#### 3.1.4 Panulirus versicolor (Latreille, 1804)

**English Name:** Painted spiny lobster

**Material examined:** Two females and one male, Bangladesh, St. Martin's Island, northeastern part of the Bay of Bengal. 20°36′49.3″N 92°19′51.6″E, ID DUZM_CR_144- DUZM_CR_144.3

**Habitat:** Shallow waters, from the sub–littoral zone down to 15 m, on coral reefs, often on the seaward edges of the reef plateau; nocturnal (Holthuis, 1991; Chan, 1998; Wahyudin et al., 2017).

**Figure 4:**
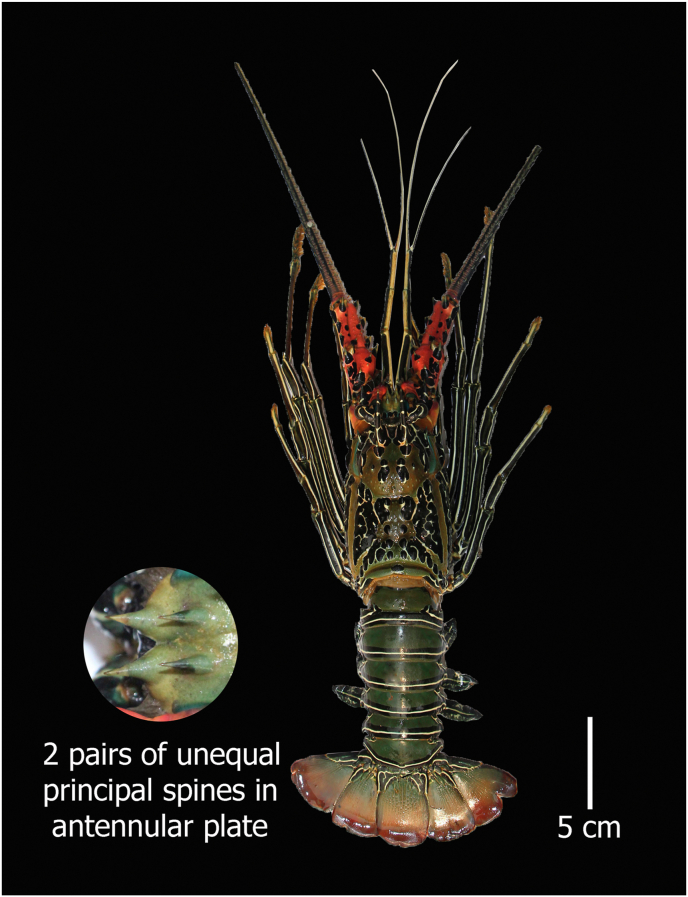
*Panulirus versicolor*, family Palinuridae, voucher ID DUZM_CR_144.3, collection date: 10-February-2021, place: St. Martin's Island.

**Characteristics:** Round-shaped carapace having numerous spines of different sizes; anterior margin with 4 regularly spaced large spines other than the frontal horns; the height of the eye is 3 times smaller than the height of the frontal horn; the antennular peduncles are smaller than antennular flagella, rostrum absent; antennular plate bearing 2 pairs of unequal and separated principal spines and separates the base of the antennae; abdominal segments without transverse grooves; 1 to 4 pereiopods without pincers (Figure 4).

**Colour:** The green–blue coloured carapace contains a distinguished pattern of blue–black patches and white lines. A transverse white band presents each abdominal segment, which is bordered by 2 black lines. Legs and antennules are covered by longitudinal stripes. Bases of the antennae contain a bright pink colour except for the antennular plate.

### 3.2 Identification key

**Guide to families**

a. The first pair of pereiopods are large, and the third pair of pereiopods have chela……Nephropidae
b. The pair of pereiopods are simple, and the third pair of pereiopods are without chela……Palinuridae

**Guide to identify species of the family palinuridae**

a. 4 equal–sized large spines in the antennular plate……………………………………………………*Panulirus homarus*
b. 1 pair of principal spines anteriorly and a second pair that is half the size of the first in antennular plate……………………………*P. ornatus*
c. Only 1 pair of equal sized large spines in the antennular plate………………………………………………………….*P. polyphagus*
d. 2 pairs of unequal and separate principal spines in the antennular plate…………………………………………………………...*P. versicolor*

### 3.3 Molecular analyses

**Figure 5:**
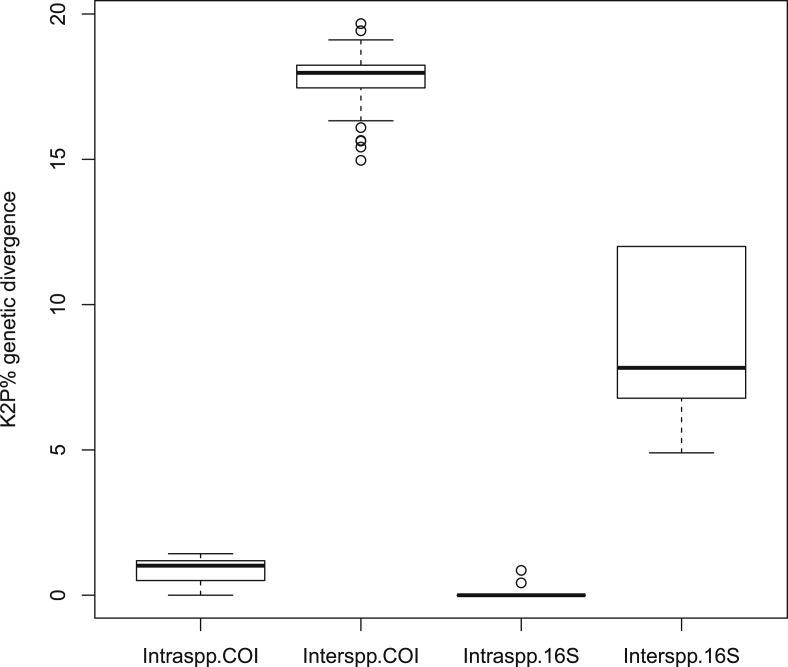
The box plot distribution of the K2P % genetic divergence within and between *Panulirus* based on COI and 16S rRNA genes.

We generated barcodes consisting of 19 partial COI and 16S rRNA sequences of the 4 *Palinurus* species belonging to the family Palinuridae (Table 1). The average 614 base pair COI sequences ranging from 410 to 679 bp had nucleotide frequencies of 32.25% T/U, 22.23% C, 25.81% A and 19.71% G. The alignment matrix of the mean 476-bp 16S rRNA ranged from 275 to 567 bp with nucleotide frequencies of 33.97% T/U, 12.96% C, 31.70% A and 21.37% G. The mean genetic divergence within and between species is summarized in Table 2 and illustrated as a box plot distribution (Figure 5). Wide barcoding gaps of 13.54% and 4.043% were observed in COI and 16S rRNA, respectively, revealing that both genes could successfully discriminate against lobster species. The maximum likelihood (ML) phylogenetic tree analysis produced three major clades, where the first group consisted of *P. polyphagus, P. homarus* and *P. ornatus*. *P. versicolor* formed a second clade, and the clade of *T. indicus* was the outgroup. Lineage support was interpreted based on bootstrap percentage (BP) [BP: 100% maximal clade support, 95% to <100% strong clade support, 75% to <95% moderate clade support, 50% to <75% weak clade support and <50% negligible clade support].

**Table 1:** GenBank Accession number of the COI and 16S rRNA sequences of Lobsters.

Sl. No.	Family	Name of the species	GB Accession No	
			COI	16S rRNA
1	Palinuridae	*Panulirus ​homarus*	MW514210-11	MW504995
2		*Panulirus ornatus*	–	MW832555
3		*Panulirus polyphagus*	MW514205-06 MW514208-09 MW832713	MW504991-92 MW504994 MW504996 MW832554
4		*Panulirus versicolor*	MW832710-12	MW832552-53

**Table 2:** Genetic divergence (percentage, K2P distance) within various taxonomic levels.

Taxonomic rank	COI (%)			16S rRNA (%)		
	Mean	Min	Max	Mean	Min	Max
Intra species	0.834 ± 0.427	0	1.43	0.107 ± 0.255	0	0.857
Inter species	17.810 ± 0.830	14.97	19.67	8.401 ± 2.547	4.90	12.00

## 4 Discussion

Morphometric key characteristics clearly identified spiny lobsters collected from the coastal region of Bangladesh (Figures 1, 2, 3, and 4), which is consistent with previous literature (Holthuis, 1991; Chan, 1998; Wahyudin et al., 2017). Among the four species of identified lobsters, *P. homarus, P. ornatus,* and *P. polyphagus* have been nationally assessed as vulnerable (VU) with *P. versicolor* assessed as endangered (EN) (IUCN Bangladesh, 2015). However, globally, all of them are considered the least concerning (LC) (IUCN, 2021). Moreover, *P. ornatus, P. polyphagus* and *P. versicolor* are listed in Schedule I of Wildlife (Conservation and Protection) Act 2012 as ‘protected animals’ (Bangladesh Gazettes, 2012). *P. polyphagus* is frequently observed in the muddy and turbid coastal belt and river mouths of Bangladesh, as this is the most suitable habitat for this species (Holthuis, 1991). Unfortunately, we observed that all of these species are indiscriminately harvested from the coastal region and trade transpired due to high prices in the domestic and overseas markets, leading this invaluable resource to be threatened with extinction.

The morphological identifications were further validated by utilizing the partial sequences of mitochondrial COI and 16S rRNA genes. The mean length of partial sequences of COI and 16S rRNA sequences generated showed AT bias, *i.e.*, high AT content in Palinuridae, similar to that in a previous study (Matzen da Silva et al., 2011). The mean K2P divergence within and between species was found to be 0.83 and 17.81, respectively, for the COI gene. In the case of Indian lobster species, the K2P divergence within and between species ranged from 0.30 to 0.70 and 15.00 to 26.80 (Jeena et al., 2016). In contrast, for 16S rRNA, the divergence within and between species was 0.11 and 8.40, respectively, which was quite similar to the value observed by Jeena et al. (2016). The calculated K2P divergence showed a significant barcoding gap, and the progressive increment in genetic divergence at a higher taxonomic level supports a marked change in genetic divergence at the species boundary. The lowest genetic divergence was observed between *P. homarus* and *P. ornatus* with a 14.97% distance in COI and 4.9% in 16S rRNA sequences. The minimum interspecific divergence greater than the 2% threshold also indicates the efficiency of markers in differentiating congeneric species. The highest divergence was found between intergenus species with 19.67% divergence between COI sequences of *P. versicolor* and *P. ornatus* from Indonesia and 12% divergence between 16S rRNA sequences of *P. polyphagus* and *P. versicolor* (Table 2).

**Figure 6:**
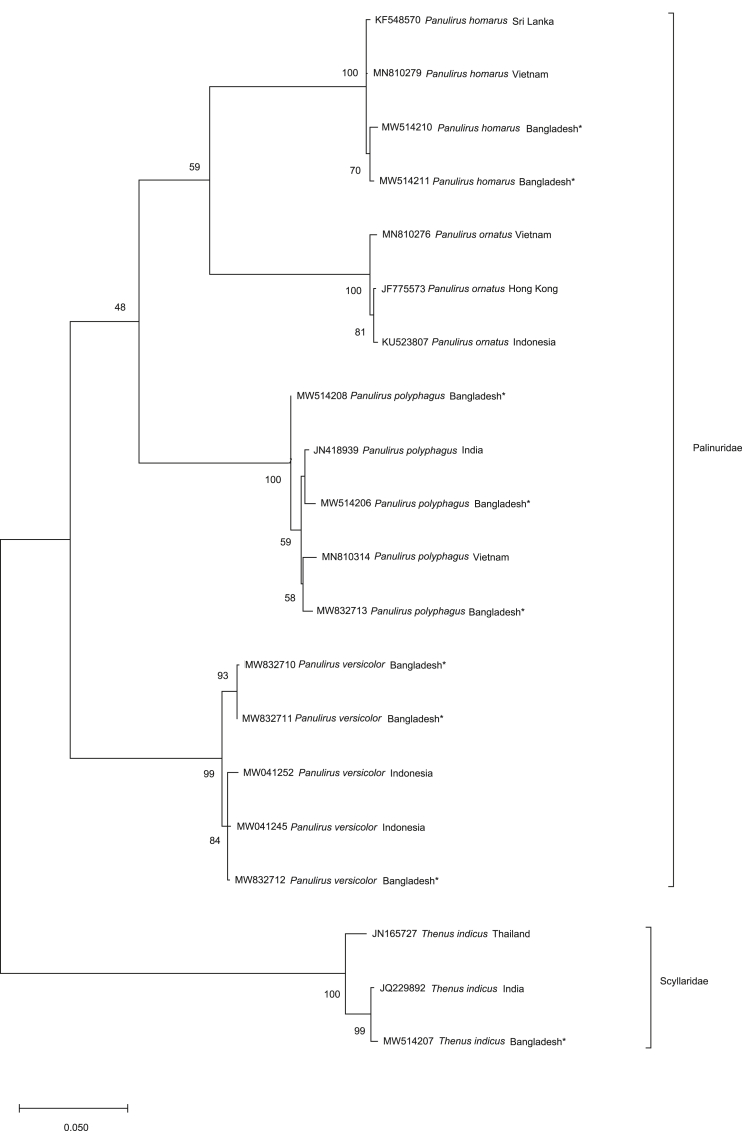
Maximum Likelihood (ML) tree showing the relationships among *Panulirus* based on COI sequences analyzed in present study with the pre-existing sequences of NCBI GenBank. (The sequences generated in this study marked as Bangladesh∗).

**Figure 7:**
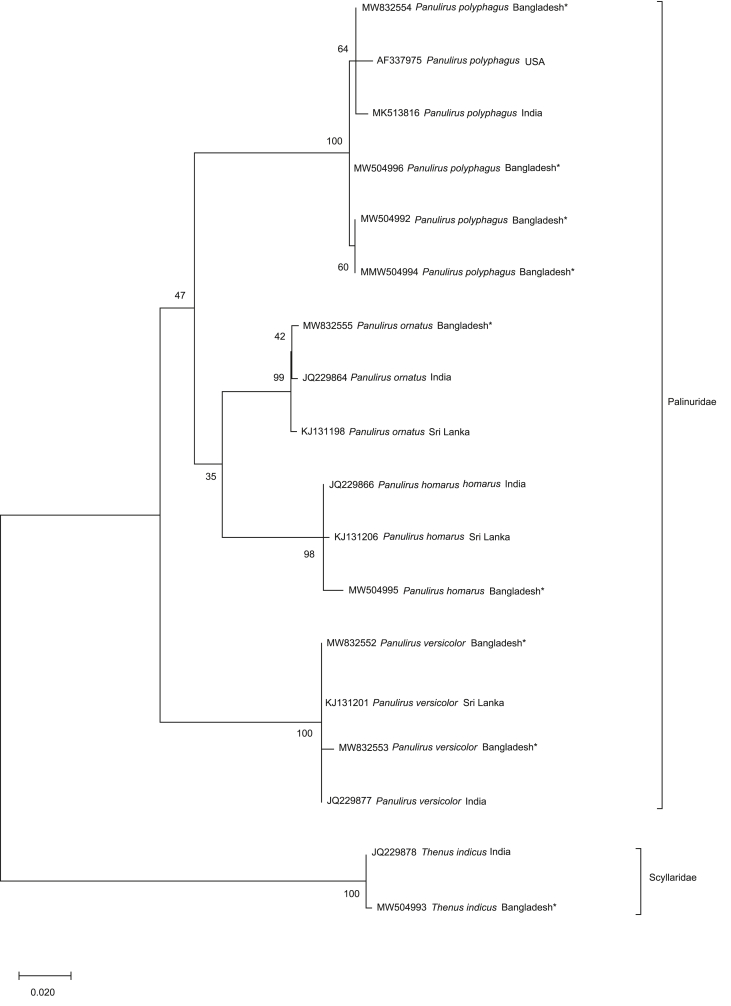
Maximum Likelihood (ML) tree showing the relationships among *Panulirus* based on 16S rRNA sequences analyzed in present study with the pre-existing sequences of NCBI GenBank. (The sequences generated in this study marked as Bangladesh∗).

The monophyletic clade of the intraspecific sequences in the phylogenetic tree constructed based on each marker gene proved the effectiveness of the COI and 16S rRNA in species delimitation. Additionally, no taxonomic deviation at the species level confirmed the authenticity of their recognition. Among the four species compared, *P. homarus* and *P. versicolor* formed close associations within species with strong clade support of 98%–100% BP in both evolutionary trees. However, the COI sequences of *P. polyphagus* formed monophyly with sequences from Vietnam and Indian sequences with 58% and 72% BP support, respectively (Figure 6), and the 16S rRNA sequences of the species grouped with the sequences from the USA and India with 64% BP (Figure 7). Additionally, the 16S rRNA sequences of *P. ornatus* formed a negligible clade with 42% BP support with species from India. The utility of molecular identification for delineating spiny lobster species found in Sri Lanka has been previously recorded by Senevirathna and Munasinghe (2013) based on partial COI sequences. The present study enables revision of the taxonomic status of *Panulirus* species residing in the geographical region of Bangladesh and their molecular characterization using partial sequences of two identification marker genes, COI and 16S rRNA, hence contributing to the global DNA barcode database.

This is the first comprehensive taxonomic description of spiny lobsters from Bangladesh, which has been further validated by two marker genes, COI and 16S rRNA. This baseline integrative approach would substantiate further taxonomic research on lobsters to understand their distribution, diversity and ecological importance in Bangladesh waters.

## Declarations

### Author contribution statement

Md. Sagir Ahmed: Conceived and designed the experiments; Analyzed and interpreted the data; Contributed reagents, materials, analysis tools or data; Wrote the paper.

Anindita Barua & Sujan Kumar Datta: Performed the experiments; Analyzed and interpreted the data; Wrote the paper.

Tonmoy Saha & Durjoy Raha Antu: Performed the experiments; Contributed reagents, materials, analysis tools or data; Wrote the paper.

Sumaiya Ahmed: Performed the experiments; Contributed reagents, materials, analysis tools or data.

### Funding statement

This work was supported by Bangladesh Academy of Sciences (BAS-USDA FI-18).

### Data availability statement

Data associated with this study has been deposited at NCBI Gen-Bank databases (https://www.ncbi.nlm.nih.gov/genbank/) under accession number MW514205-06, MW514208-11, MW832710-13, MW504991-92, MW504994-96, and MW832552-55.

### Declaration of interests statement

The authors declare no conflict of interest.

### Additional information

No additional information is available for this paper.
